# Renal sympathetic nerve activity after catheter-based renal denervation

**DOI:** 10.1186/s13550-018-0360-1

**Published:** 2018-01-26

**Authors:** Linn C. Dobrowolski, Daan W. Eeftinck Schattenkerk, C. T. Paul Krediet, Peter M. Van Brussel, Liffert Vogt, Frederike J. Bemelman, Jim A. Reekers, Bert-Jan H. Van Den Born, Hein J. Verberne

**Affiliations:** 1 Department of Internal Medicine - Nephrology and Kidney Transplantation, Academic Medical Center at the University of Amsterdam, Amsterdam, the Netherlands; 2 Department of Internal Medicine - Vascular Medicine, Academic Medical Center at the University of Amsterdam, Amsterdam, the Netherlands; 3 Department of Cardiology, Academic Medical Center at the University of Amsterdam, Amsterdam, the Netherlands; 4 Department of Radiology and Nuclear Medicine, F2-238 Academic Medical Center, University of Amsterdam, Meibergdreef 9, 1105 AZ Amsterdam, The Netherlands

**Keywords:** Renal catheter ablation, Hypertension, Radionuclide imaging, Renal nerves, Sympathetic nerve activity

## Abstract

**Background:**

Catheter-based renal sympathetic denervation (RDN) has been considered a potential treatment for therapy resistant hypertension (RHT). However, in a randomized placebo-controlled trial, RDN did not lead to a substantial blood pressure (BP) reduction. We hypothesized that variation in the reported RDN efficacy might be explained by incomplete nerve disruption as assessed by renal ^123^I–*meta*-iodobenzylguanidine (^123^I–*m*IBG) scintigraphy.

**Methods:**

In 21 RHT patients (median age 60 years), we performed ^123^I–*m*IBG scintigraphy before and 6 weeks after RDN. Additionally, we assessed changes in BP (24 h day, night, and average), plasma- and urinary-catecholamines and plasma renin activity (PRA) before and after RDN. Planar scintigraphy was performed at 15 min and 4 h after ^123^I–*m*IBG administration. The ratio of the mean renal (specific) counts vs. muscle (non-specific) counts represented ^123^I–*m*IBG uptake. Renal ^123^I–*m*IBG washout was calculated between 15 min and 4 h.

**Results:**

After RDN office-based systolic BP decreased from 172 to 153 mmHg (*p* = 0.036), while diastolic office BP (*p* = 0.531), mean 24 h systolic and diastolic BP (*p* = 0.602, *p* = 0.369, respectively), PRA (*p* = 0.409) and plasma catecholamines (*p* = 0.324) did not significantly change post-RDN. Following RDN, ^123^I–*m*IBG renal uptake at 15 min was 3.47 (IQR 2.26–5.53) compared to 3.08 (IQR 2.79–4.95) before RDN (*p* = 0.289). Renal ^123^I–*m*IBG washout did not change post-RDN (*p* = 0.230). In addition, there was no significant correlation between the number of denervations and the renal ^123^I–*m*IBG parameters.

**Conclusions:**

No changes were observed in renal ^123^I–*m*IBG uptake or washout at 6 weeks post-RDN. These observations support incomplete renal denervation as a possible explanation for the lack of RDN efficacy.

## Background

Reduction of sympathetic nerve activity by catheter-based renal sympathetic denervation (RDN) has raised considerable attention as a new treatment modality for resistant hypertension (RHT). This interest was fueled by the promising results of RDN in the initial open label studies Symplicity HTN-1 and HTN-2 [1–3]. However, the recent randomized sham-controlled Symplicity HTN-3 trial did not show a difference in blood pressure (BP) lowering efficacy between RDN and sham treatment [4]. One of the potential causes for the lack of efficacy might be the failure of the RDN procedure to sufficiently ablate renal sympathetic nerves. Yet, a routine technique to measure the extent of renal denervation is lacking and potential causes of insufficient denervation remain hypothetical.

^123^I–*meta-*iodobenzylguanidine (^123^I–*m*IBG) scintigraphy offers the possibility to evaluate organ specific presynaptic sympathetic nerve activity. *m*IBG is an analogue of the “false” neurotransmitter guanetidine, a potent neuron blocking agent that acts selectively on sympathetic nerves. *m*IBG follows similar uptake mechanisms as norepinephrine: as such, *m*IBG-uptake enables assessment of the intactness and density of the neural tissue. Radiolabelling of *m*IBG with ^123^Iodide enables scintigraphic assessment. ^123^I–*m*IBG organ uptake and washout reflect sympathetic activity. Uptake of ^123^I–*m*IBG reflects the density and functional intactness of the neural tissue within the organ, whereas washout is thought to reflect sympathetic activity [5, 6]. Previously, we tested this technique for visualizing renal sympathetic innervation by showing its ability to detect changes in sympathetic innervation during kidney allograft re-innervation [7].

Based on the inter-individual variation in BP response after RDN, we hypothesized that there is a wide variability in kidney sympathetic denervation following RDN. Secondly, we hypothesized that changes in renal sympathetic activity would relate to changes in BP and neurohormonal activity following RDN. Against this background, we examined changes in renal ^123^I–*m*IBG uptake and washout in RHT patients before and after RDN treatment.

## Methods

From July 2011 to December 2013, we performed a prospective observational study using ^123^I–*m*IBG scintigraphy as a parameter of renal sympathetic activity in patients with RHT undergoing RDN. Objectives were to compare measures of renal ^123^I–*m*IBG uptake (uptake at 15 min and washout between 15 min and 4 h) on planar and single photon emission computed tomography-CT (SPECT-CT) images, changes in office based BP and ambulatory BP measurements (ABPM) and neurohormonal activation before and 6 weeks after RDN.

### Patients

In the present study, we enrolled 21 consecutive patients aged 40–70 years with a clinical indication for RDN because of therapy resistant hypertension defined as a mean daytime BP ≥ 150/100 mmHg despite the use of three or more anti-hypertensive drugs including or with intolerance to a diuretic [8]. Secondary causes of hypertension (e.g., renal artery stenosis, pheochromocytoma, primary aldosteronism, and hyper- or hypothyroidism) and abnormal renal artery anatomy, including the presence of accessory renal arteries, were ruled out prior to the intervention. Patients with renal insufficiency (estimated glomerular filtration rate (eGFR) < 45 mL/min/1.73 m^2^) or proteinuria (> 1 g/24 h) or having a pacemaker, implantable cardioverter-defibrillator (ICD), atrial fibrillation, or type 1 diabetes mellitus were excluded.

Antihypertensive treatment was performed according to international guidelines and included instructions on dietary sodium restriction, physical activity, and instructions to remain compliant to antihypertensive medication [8, 9]. Six weeks prior to the first measurements, patients were screened to assess eligibility for study participation. Patients were deemed eligible for study participation if they were at least 3 weeks on stable BP lowering medication prior to the first study visit. BP lowering medication was kept unchanged throughout the study until the final visit 6 weeks after RDN.

When fully informed and willing to participate, patients were asked to provide written informed consent. Six weeks hereafter, office BP and ABPM were measured. Patients were required to maintain the same antihypertensive drug regimen throughout study participation. This study was a part of a larger effort to assess the sympaticolytic potential of RDN with the predetermined idea to assess the effects of RDN on renal ^123^I–*m*IBG uptake and washout.

For reference, we used data of five patients (aged 39–66 years) in whom ^123^I–*m*IBG was performed of the kidney allograft after recent kidney transplantation (0.1 to 1.5 years after transplantation), whose detailed characteristics are described elsewhere [7]. In summary, all these surgically denervated kidneys functioned well with creatinine clearance rates (calculated from 24 h urine collections) ranging from 54 to 128 ml/min. As a negative control, we also included ^123^I–*m*IBG data from a patient with complete renal denervation after autologous kidney transplantation for renal artery stenosis [10]. Although ^123^I–*m*IBG is primarily cleared via the kidneys, we have shown that both the cardiac as well as the renal ^123^I–*m*IBG parameters (i.e., late heart-to-mediastinal ratio, renal uptake, and renal washout) are not influenced by kidney function [7, 11].

### Study protocol

The study protocol met the ethical guidelines of the Declaration of Helsinki (originally adopted by the 18th WMA General Assembly, Helsinki, Finland, June 1964 and last amended in Fortaleza, Brazil 2013) and was approved by the local ethics committee of the Academic Medical Center at the University of Amsterdam (number NL.36755.018.11). All patients gave oral and written informed consent before study inclusion.

### Renal sympathetic denervation procedure

The renal denervation procedure was performed via the femoral artery approach by a single highly experienced interventional radiologist (JAR) with > 5 RDN procedures before this study was initiated. RDN was performed by use of radiofrequency energy delivered by the Symplicity renal-denervation catheter (Medtronic Inc., Santa Rosa, California, USA). Prior to the procedure, midazolam 1.0 mg and metoclopramide 10 mg was given intravenously. After inserting a 6 F introducer sheath in the right femoral artery, the guiding catheter was introduced in the aorta and an aortagram was made. The guiding catheter was advanced in the right and left renal artery in no pre-specified order. The denervation catheter was introduced in the renal artery via the guiding catheter. After nitroglycerine 0.2 mg and fentanyl 0.02 mg intravenously, catheter ablations were performed in a helical pattern with the goal of at least 4–6 ablations per renal artery to cover each short axis transaxial quadrant, according to the user’s instruction of the device. No peri-procedural complications occurred.

### Blood pressure monitoring

At baseline and 6 weeks after RDN 24 h ABPM was performed using the Spacelabs 90,217 ABPM monitoring device (Spacelabs Healthcare, Issaquah, Washington, USA). During day time between 06.00 am and 23.00 pm, measurements were performed every 15 min and at night-time (i.e., 23.00 pm and 6.00 am) every 30 min. BP readings were accepted when the success rate of the measurements was minimally 70% per 24 h. Patients were blinded to their BP readings. Instructions were given to continue usual daily activities during 24 h of BP recording, but avoiding strenuous exercise. Office brachial BP using appropriate cuff-sizes was measured with a validated semi-automated oscillometric device (Omron 705it, Omron Healthcare Europe BV, Hoofddorp, The Netherlands), while seated and after 5 min rest in a quiet room, three times at 1 min intervals by a trained research assistant or physician. The mean of the last two measurements was recorded as representative of office brachial BP. No BP measurements were performed in the kidney transplant recipient group.

### Blood and urine analysis

Plasma renin activity (PRA) (μgA1/L/h) was analyzed using radioimmunoassays. Urine and plasma epinephrine, norepinephrine (NE), metanephrine, and normetanephrine were analyzed using liquid chromatography-mass spectrometry. Epinephrine and NE and were obtained in supine as well as after 5 min in standing position. The delta of supine minus standing position was calculated. Urinary sodium excretion (mmol/24 h), urine creatinine (μmol/L), was calculated from 24 h urine collections obtained before and 6 weeks post-RDN.

### ^123^I–*m*IBG scintigraphy

The protocol of the renal ^123^I–*m*IBG scintigraphy has been previously described [7]. In summary, 2 h prior to the administration of 185 MBq (5 mCi ± 10%) ^123^I–*m*IBG (AdreView™, GE Healthcare, Eindhoven, the Netherlands) patients received 100 mg potassium-iodide to block thyroid uptake of “free” ^123^I. In addition subjects were given a single oral dose of furosemide retard 60 mg to promote the urinary excretion of ^123^I–*m*IBG. No specific instructions on fluid intake were given to enhance excretion of ^123^I–*m*IBG. Anterior and posterior planar semi-whole body images were performed at 15 min and 4 h after administration of ^123^I–*m*IBG. A vial with a reference amount of radioactivity of ^123^I was included in the planar images. Additionally, at 4 h post-injection (p.i.), SPECT-CT (low dose) was performed. The CT-images were used for an adequate anatomical registration of ^123^I–*m*IBG uptake.

Since we recently showed that uptake at 15 min p.i. of ^123^I–*m*IBG and washout between 15 min and 4 h can detect renal sympathetic reinnervation over time after transplantation, we report in this study the ^123^I–*m*IBG uptake on the 15 min p.i. images and analyzed the mean counts/pixel for calculation of washout between 15 min and 4 h [7].

### ^123^I–*m*IBG imaging procedures

The planar images were acquired with a 20% energy window centered at 159 keV, using medium-energy collimators. Anterior and posterior planar semi-whole body acquisitions were used to create geometrical mean images.

### ^123^I–*m*IBG image analysis

An investigator (LCD) analyzed the geometric mean (GM) planar images (Hybrid Viewer™, Hermes Medical Solutions, Stockholm, Sweden) by manually drawing regions of interest (ROI) for kidneys, muscle (*M. quadriceps* femoris), and the ^123^I vial. A predefined and fixed ROI for the muscle (50 pixels) was used for all patients.

We analyzed the counts of the left kidney only since scatter or overlay of the liver with a high uptake of ^123^I–*m*IBG resulted in poor delineation of the right kidney. Mean counts per pixel per ROI were used to calculate ^123^I–*m*IBG uptake: the relative uptake between kidney (specific) versus muscle (nonspecific) quantifies neural uptake of ^123^I–*m*IBG and reflects neuron function that results from ^123^I–*m*IBG uptake, storage and release. These can be derived using mean counts from the 15 min and 4 h p.i. GM images and the 4 h p.i. ^123^I–*m*IBG SPECT-CT images. Washout (WO) between 15 min and 4 h p.i. based on GM images reflects sympathetic activity and was calculated from the kidney-to-muscle ratio between 15 min and 4 h p.i.. Formulas to calculate uptake and washout were

$$ \mathrm{Relative}\ \mathrm{uptake}=\frac{\mathrm{kidney}\ \left(\mathrm{specific}\right)-\mathrm{muscle}\ \left(\mathrm{non}-\mathrm{specific}\right)}{\mathrm{muscle}\ \left(\mathrm{non}-\mathrm{specific}\right)} $$

$$ \mathrm{Washout}=\frac{\left(\ \frac{\mathrm{uptake}\ \mathrm{kidney}\ 15\ \min }{\mathrm{uptake}\ \mathrm{muscle}\ 15\min}\right)-\left(\frac{\mathrm{uptake}\ \mathrm{kidney}\ 4\ \mathrm{h}\ }{\mathrm{uptake}\ \mathrm{muscle}\ 4\ \mathrm{h}}\right)\ }{\left(\ \frac{\mathrm{uptake}\ \mathrm{kidney}\ 15\ \min }{\mathrm{uptake}\ \mathrm{muscle}\ 15\min}\right)}\mathrm{x}100\% $$

The percentage uptake of the injected dosage of ^123^I–*m*IBG was calculated using the actual injected dose and mean counts per pixel in relation to the activity in the ^123^I–vial. Washout [WO) in the left kidney was calculated from 15 min and 4 h images using skeletal muscle as a reference.

A secondary analysis was focused on the SPECT-CT images. In this method, the transverse CT images were used to optimize anatomical delineation of the kidney contours. The main advantage of this method is the availability of anatomical information obtained from the low dose CT, allowing for a superior delineation of kidneys and subsequently a potential better estimation of the renal ^123^I–*m*IBG uptake. ROIs were drawn on the CT-images along the contours of kidney cortices, excluding the calyces. ROIs were then fused into volumes of interest (VOIs) and copied to the co-registered SPECT. Mean counts/voxel expressed ^123^I–*m*IBG uptake. VOIs in muscle served as background activity.

Based on the difference in ^123^I–*m*IBG uptake, we divided patients with a positive change in ^123^I–*m*IBG uptake, i.e., indicating an increase in ^123^I–*m*IBG uptake or washout and those with a negative change, i.e., a decrease in ^123^I–*m*IBG uptake or washout after RDN.

### Statistical analysis

This study was part of a larger effort to study sympatholytic effects of RDN. The sample size has been described elsewhere [12]. Data are presented as medians and interquartile ranges (IQR with 25 and 75 percentiles) and comparisons were performed by non-parametrical tests (Wilcoxon signed rank tests as well as the Mann–Whitney *U* test). *P* values below 0.05 were considered statistically significant. All analyses were performed using IBM SPSS Statistics software for Windows version 21.0 (IBM Corp. Armonk, New York, USA).

## Results

### Baseline characteristics

We studied 21 patients with therapy resistant hypertension (Table 1). The majority of patients were male (71% with a median 60 years) and Caucasian (76%). Median body mass index was 28.0 kg/m^2^ (24.8–30.5 kg/m^2^). Type 2 diabetes mellitus was present in 33% and left ventricular hypertrophy, according to electrocardiography voltage criteria, was present in 29% of the patients. A history of a cardiovascular disease (coronary artery disease, angina pectoris, heart failure, stroke, peripheral arterial disease) was present in 48% of the study participants.

**Table 1:** Characteristics of patients treated with RDN (*n* = 21)

Characteristics of patients treated with RDN (*n* = 21)	
Male, *n* [%)	15 (71.4)
Age at intervention (years)	60 [53–70]
Caucasian ethnicity, *n* (%)	16 (76.2)
Weight (kg)	88.0 [69.5–99.5]
Body mass index (kg/m^2^)	28.0 [24.8–30.5]
Type 2 diabetes mellitus, *n* (%)	7 (33.3)
Left ventricular hypertrophy, *n* (%)	6 (28.6)
History of any cardiovascular event, *n* (%)	10 (47.7)
Proteinuria (g/L/24 h)	0.10 [0.07–0.20]
Macroalbuminuria, *n* (%)	2 (9.5)
*N*^0^ of denervation pulses left renal artery	4.3 ± 0.6
*N*^0^ of denervation pulses right renal artery	4.2 ± 0.5

Data are presented either as number (*n*) and percentage (%) or as medians and interquartile ranges (IQR with 25 and 75 percentiles)

### Renal ^123^I–*m*IBG uptake and washout in the left kidney

**Fig. 1:**
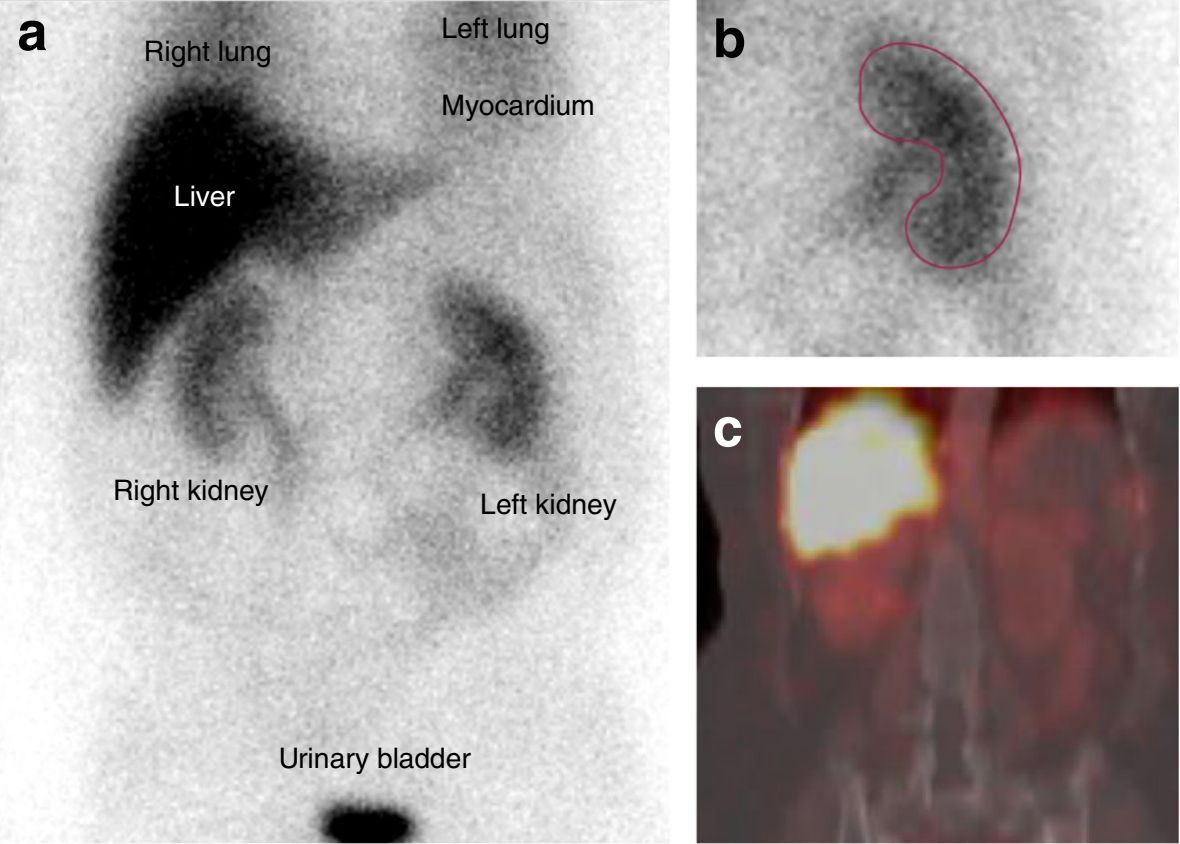
Anterior planar and SPECT-CT ^123^I–*m*IBG scintigraphy. The planar image (**a**) shows clear uptake of ^123^I–*m*IBG uptake in various organ: liver, urinary bladder and evident uptake of ^123^I–*m*IBG in both kidneys. **b** Shows the ROI on the planar image of the left kidney trying to exclude any pelvic activity. **c** Shows a coronal slice of the SPECT-CT showing the proximity of the liver to the right kidney. The proximity of the liver to the right kidney can also be appreciated on the planar images (**a**). Thereby, both planar and SPECT images illustrate the possible impact of liver activity on parameters of ^123^I–*m*IBG uptake in the right kidney

**Fig. 2:**
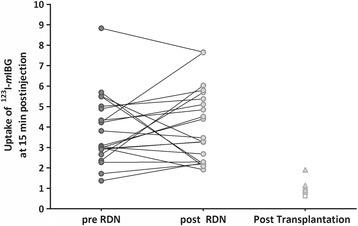
Change in renal uptake of ^123^I–*m*IBG after RDN. The planar derived mean relative uptake of ^123^I–*m*IBG of the left kidney at 15 min p.i. did not change significantly from pre RDN 3.08 (2.79–4.95) to post RDN 3.47 (2.26–5.53), *p* = 0.289. Included on the right side of the figure is depicted the relative kidney uptake of ^123^I–*m*IBG in a group patients with kidney transplantations, serving as a reference

Renal ^123^I–*m*IBG uptake was evident in all patients (Fig. 1). The planar derived mean relative uptake of ^123^I–*m*IBG of the left kidney at 15 min p.i. did not change significantly from pre RDN 3.08 (2.79–4.95) to post RDN 3.47 (2.26–5.53), *p* = 0.289 (Table 2). Figure 2 represents pre vs. post RDN ^123^I–*m*IBG uptake at 15 min p.i. including recently transplanted kidneys as controls.

**Table 2:** Pre and post RDN differences in quantifications of ^123^I–*m*IBG uptake (*n* = 21)

	PRE-RDN	POST-RDN	*p* value
Planar GM images			
Uptake 15 min	3.08 [2.79–4.95]	3.47 [2.26–5.53]	0.289
Uptake 4 h	1.64 [1.44–1.98]	1.52 [1.12–2.27]	0.876
% Injected dose 15 min*	17.88 [17.88–21.75]	15.43 [13.73–22.13]	0.881
% Injected dose 4 h*	8.91 [8.91–13.52]	9.37 [7.20–12.35]	0.681
Washout 15 min-4 h (%)	41.53 [28.26–56.25]	42.69 [35.02–56.16]	0.230
SPECT-CT images			
Uptake CT 4 h	1.41 [0.95–1.86]	1.07 [0.73–1.69]	0.526

Data are presented as medians with interquartile ranges (IQR 25–75%). RDN = Renal denervation, GM = geometric mean images, with muscle as background, SPECT = single photon emission computed tomography. n.a. = not available, *data from *n* = 20 patients since in one patient a ^123^I–vial was not included during the scintigraphy and therefore the percentage of injected dose ^123^I–*m*IBG could not be calculated

The percentage uptake of the injected dosage of ^123^I–*m*IBG in the left kidneys showed a non-significant decrease after RDN from 17.8 to 15.4% (delta − 13%, *p* = 0.881). Washout rate between 15 min and 4 h p.i. was 41.5% before and 42.7% after RDN, *p* = 0.230. The SPECT derived uptake at 4 h decreased non-significantly after RDN (1.41 to 1.07, *p* = 0.526). None of the renal uptake or washout parameters were correlated with kidney function (data not shown).

### Number of denervations and renal ^123^I–*m*IBG uptake and washout

No significant correlation was found between the number of denervations (left renal artery (4.3 ± 0.6), right renal artery 4.2 ± 0.5), and renal uptake of ^123^I–*m*IBG in the left kidney at either 15 min (*R* = − 0.27, *p* = 0.243), 4 h p.i. (*R* = − 0.37, *p* = 0.103) or ^123^I–*m*IBG washout (*R* = 0.05, *p* = 0.837).

### Effect of RDN on blood pressure, PRA, and catecholamines

Table 3 shows the effect of RDN on blood pressure and catecholamines. RDN resulted in a significant decrease in systolic office BP (*p* = 0.036), without reducing diastolic BP (*p* = 0.531). Systolic and diastolic daytime ABPM were not significantly different after denervation. Neither antihypertensive medication nor sodium intake, as inferred from urinary sodium excretion, were significantly different between pre vs. post-RDN (Table 2).

**Table 3:** Blood pressure, kidney function and catecholamines

	PRE-RDN	POST-RDN	*p* value
Parameters			
*Blood pressure*			
Office based systolic (mmHg)	172.0 [162.0–185.0]	153.0 [140.0–178.0]	0.036
Office based diastolic (mmHg)	97 [90.5–112.5]	90.0 [81.5–100.5]	0.531
ABPM daytime systolic (mmHg)	166.0 [157.0–179.5]	165.0 [141.5–186.0]	0.578
ABPM daytime diastolic (mmHg)	98.0 [87.0–108.0]	93.0 [83.0–99.5]	0.409
ABPM night time systolic (mmHg)	151.0 [133.5–158.5]	145.0 [125.0–165.5]	0.490
ABPM night time diastolic (mmHg)	84.0 [75.5–90.0]	80.0 [71.0–91.5]	0.640
ABPM average systolic (mmHg)	160.0 [150.5–173.0]	157.0 [138.5–174.0]	0.602
ABPM average diastolic (mmHg)	93.0 [83.5–100.5]	92.0 [80.0–94.5]	0.369
*Antihypertensive drugs*			
Number of antihypertensive drugs	4.6 ± 1.3	4.4 ± 1.4	0.157
3 classes, *n* (%)	5 (23.8)	7 (33.3)	
4 or more classes, *n* (%)	16 (76.2)	14 (66.7)	
*Kidney function*			
Creatinine serum (μmol/L)	94.0 [76.5–107.5]	89.0[73.5–113.5]	0.369
eGFR (ml/min/1.73 m2)	60.7[48.5–101.9]	64.6 [48.0–99.9]	0.218
Proteinuria (g/L/24 h)	0.10 [0.07–0.20]	0.11 [0.07–0.26]	0.722
Sodium urine (mmol/24 h)	161 [102–203]	128 [90–161]	0.230
*(Neuro) endocrine activity*			
Plasma renin activity (μg/A1/L/h)	1.70 [0.95–3.20]	1.0 [0.60–1.68]	0.409
Epinephrine supine, plasma (nmol/L)	0.12 [0.05–0.23]	0.10 [0.05–0.17]	0.780
Norepinephrine supine, plasma (nmol/L)	2.43 [1.32–3.78]	2.76 [1.49–4.02]	0.324
Epinephrine urine (nmol/24 h)	27.5 [14.5–33.8]	26.0 [18.0–38.0]	0.551
Norepinephrine urine (nmol/24 h)	268.5 [137.5–495.0]	308.5 [237.5–479.3]	0.245
Metanephrine urine (nmol/24 h)	0.78 [0.49–1.05]	0.68 [0.50–1.02]	0.506
Normetanephrine urine (nmol/24 h)	2.13 [1.73–3.37]	2.53 [1.74–3.02]	0.911

At baseline, plasma and urine catecholamine levels were within reference values. Plasma epinephrine and NE did not change (*p* = 0.780 and *p* = 0.324, respectively) nor did the 24 h urinary excretion of metanephrine (*p* = 0.506) and normetanephrine (*p* = 0.911) following RDN (Table 3).

### Renal ^123^I–*m*IBG uptake and washout and blood pressure, PRA, and catecholamines

**Fig. 3:**
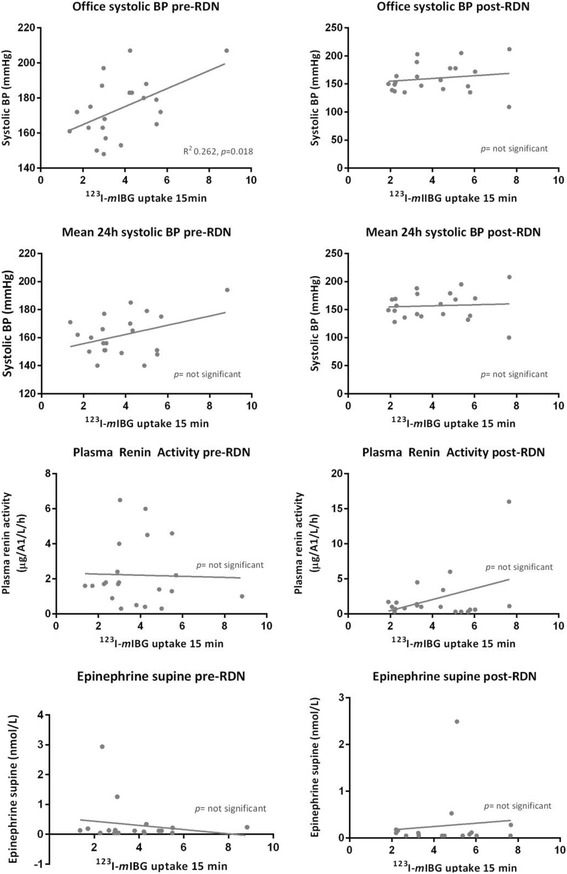
Renal ^123^I–*m*IBG uptake in relation to blood pressure and biochemistry data. There was only a significant correlation between renal ^123^I–*m*IBG uptake and office systolic BP (*p* = 0.018). No other correlations were found between any of the renal ^123^I–*m*IBG uptake and washout parameters and blood pressure, PRA or catecholamines

Except for the correlation between renal ^123^I–*m*IBG uptake and office systolic BP (*p* = 0.018), no correlations were found between any of the renal ^123^I–*m*IBG uptake and washout parameters and blood pressure, PRA or catecholamines (Fig. 3).

#### Subgroup analyses

**Fig. 4:**
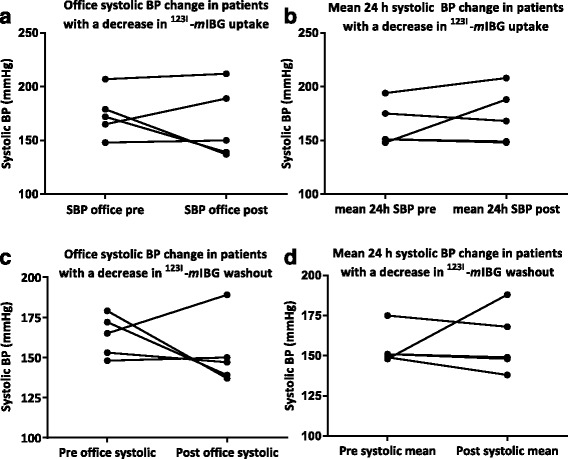
**a** Pre- and post-RDN office systolic BP change in patients with the largest decrease in ^123^I–*m*IBG uptake. **b** Pre- and post-RDN mean 24 h systolic BP in patients with the largest decrease in ^123^I–*m*IBG uptake. **c** Pre- and post-RDN office systolic BP change in patients with the largest decrease in ^123^I–*m*IBG washout in patients with the largest decrease in ^123^I–*m*IBG uptake. **d** Pre- and post-RDN mean 24 h systolic BP change in patients with the largest decrease in ^123^I–*m*IBG washout in patients with the largest decrease in ^123^I–*m*IBG washout

Patients with the largest decrease in ^123^I–*m*IBG uptake at 15 min (i.e., delta ≤ − 1.0) (*n* = 5) and patients with the largest increase in ^123^I–*m*IBG uptake at 15 min (i.e., delta of ≥1.0) (*n* = 5) did not differ in ABPM, kidney function, or catecholamine levels after RDN (Fig. 4 and Table 4), nor did the patients with the largest change (i.e., both increased and decreased) in ^123^I–*m*IBG uptake differ in baseline characteristics from the patients without these changes in ^123^I–*m*IBG uptake (data not shown).

**Table 4:** Parameters pre-RDN in patients with a positive delta (i.e., increase in ^123^I–*m*IBG uptake at 15 min) versus patients with a negative delta (i.e., decrease in ^123^I–*m*IBG uptake at 15 min) after RDN

Baseline parameters (pre-RDN)			
	Increase (*n* = 14)	Decrease (*n* = 7)	*p* value
*Demography*			
Male *n*	10	5	0.701
Age (years)	56.4 [48.6–63.3]	67.0 [60.5–67.6]	0.062
BMI (kg/m^2^)	26.98 [24.52–28.96]	30.02 [27.4725–31.7451]	0.205
Left ventricular hypertrophy, *n*	4	2	0.686
CVD, *n*	5	5	0.140
Type 2 diabetes mellitus, *n*	3	4	0.127
Ethnicity (Caucasian), *n*	11	5	0.557
*Blood pressure*			
Antihypertensives, *n*	4.6 ± 1.2	4.4 ± 1.6	0.590
Office systolic BP (mmHg)	177.5 [162.5–187.3]	168.0 [153.0–179.0]	0.370
Office diastolic BP	104.0 [92.0–116.5]	95.0 [76.0–102.0]	0.101
ABPM daytime systolic BP	171.50[157.5–181.8]	159.00 [154.0–173.0]	0.433
ABPM daytime diastolic BP	100.5 [93.5–112.5]	90.0 [81.0–101.0]	0.126
ABPM night time systolic BP	153.0 [135.0–158.3]	139.0 [132.0–180.0]	0.550
ABPM night time diastolic BP	86.5 [77.0–91.3]	77.0 [71.0–87.0]	0.145
ABPM average systolic BP	163.5 [154.5–172.5]	151.0 [149.0–175.0]	0.411
ABPM average diastolic BP	95.5 [88.0–105.0]	85.0 [80.0–96.0]	0.101
^*123*^ *I–mIBG parameters*			
Washout GM 15 min - 4 h	35.463 [25.68–47.36]	59.365 [38.73–62.39]	0.014
GM uptake 15 min	2.9590 [2.3403–4.26]	5.4906 [3.03–5.69]	0.017
GM uptake 4 h	1.84 [1.17–2.14]	1.53 [1.45–1.64]	0.456
CT uptake 4 h	1.33 [0.84–2.07]	1.56 [1.26–1.87]	0.552
*Kidney function*			
eGFR (ml/min/1.73 m2)	63.6 [49.4–96.1]	53.3 [48.4–138.3]	0.765
Serum creatinine (μmol/L)	91.0 [77.8–112.5]	98.0 [67.0–104.0]	0.852
Sodium urine (mmol/24 h)	150.80 [104.40–200.55]	170.1 [71.5–275.4]	1.000
*(Neuro) endocrinology*			
Plasma renin activity (μg/A1/L/h)	1.65 [0.78–1.95]	2.20 [1.00–4.60]	0.370
Epinephrine supine, plasma (nmol/L)	0.12 [0.05–0.15]	0.17 [0.05–0.50]	0.554
Norepinephrine supine, plasma (nmol/L)	2.72 [1.22–3.96]	2.22 [1.71–2.89]	0.837
Epinephrine urine (nmol/24 h)	26.00 [10.75–36.25]	28.00 [20.75–32.25]	0.903

Data are presented as medians with interquartile ranges (IQR 25–75%). GM = geometric mean images, with muscle as background, SPECT = single photon emission computed tomography, RDN = Renal denervation GM = geometric mean

In patients with the largest decrease in washout (i.e., delta ≤ − 5.0) (*n* = 5), there were no changes in BP measurements, neither in catecholamines nor in kidney function (data not shown). In patients with the largest decrease in ^123^I–*m*IBG washout, only the 24 h urine metanephrine was significantly higher at baseline compared to patients with the largest increase in washout after RDN (*p* = 0.045). In patients with the largest increase in ^123^I–*m*IBG washout (i.e. delta ≥5.0) (*n* = 10) there was a difference in office systolic BP only (pre vs. post RDN median 181.5 vs. 158.0 mmHg, *p* = 0.05), while diastolic BP did not change. In addition this subgroup did not show significant changes in ABPM, kidney function or catecholamines after RDN (data not shown).

No correlations were found between any of the renal ^123^I–*m*IBG uptake parameters and BP measurements (data not shown).

## Discussion

In the present study we were unable to demonstrate that treatment with RDN results in significant changes in renal ^123^I–*m*IBG uptake and washout. These data suggest that RDN does not significantly alter renal sympathetic tone and does not sufficiently denervate renal sympathetic nerves. This is further supported by the finding that ABPM and biochemical markers of sympathetic nerve activity remained unchanged after RDN, while the reduction in office BP was similar compared to Symplicity HTN-1 and HTN-2 [1, 2]. The absence of consistent changes in ^123^I–*m*IBG uptake and washout as well as the lack of a sustained BP decrease after RDN suggests that the present RDN technique fails to achieve adequate denervation of the kidneys. The degree of renal sympathetic nerve disruption required for inducing a sustained BP response remains unclear, but likely falls short with the current RDN technique. The lack of efficacy may be related to the number of ablations, since in a subset of patients of Symplicity HTN-3 a more profound BP decrease was observed in patients with more ablations, suggesting a relation between the quantity of ablations and the BP lowering effects [4]. This effect, however, was also observed in patients receiving sham treatment. We found no association between the number of ablations and renal ^123^I–*m*IBG uptake or washout, while the number of denervations in our study was similar to the Symplicity HTN-1 and HTN-2 trials that demonstrated a significant decrease in office BP [1, 2].

In a post-mortem study of a patient who received RDN it was shown that nerves in the (peri-) adventitial parts of the renal artery were unaffected, indicating that interruption of the nerve fiber continuity had not been successful [13]. This suggests that the ablation pulse may not be sufficient to generate adequate denervation of renal sympathetic nerves [14]. A previous study using NE spill-over to assess the effect of the nerve fiber continuity had not been successful [13]. In another study, using NE spill-over to assess the effect of RDN on renal sympathetic activity in 10 patients with resistant hypertension showed that RDN reduced NE spill-over by 47% (95% CI 28–65%) [15]. In the present study, we could not replicate these findings.

Besides lack of procedural effectiveness, this discrepancy could also be explained by differences in population characteristics or technical shortcomings of ^123^I–*m*IBG scintigraphy. The patients in our study were however fully comparable to the populations studied in Symplicity HTN-1 and Symplicity HTN-2.

Although, we used ABPM instead of office BP to include patients with resistant hypertension, baseline office BP in our study and the number of BP lowering drugs were comparable to that observed in Symplicity HTN-1 and Symplicity HTN-2. In addition, office BP was reduced to a similar extent with a decrease of 29 mmHg for systolic office BP following RDN. All other baseline parameters of our study population were similar to that of previous studies [1, 2, 4]. In kidney transplant recipients we recently showed that uptake at 15 min p.i. of ^123^I–*m*IBG and washout is correlated with time after transplantation independent of kidney graft function [7]. This suggests that renal ^123^I–*m*IBG scintigraphy can be used to assess differences in renal innervation.

We previously showed that cardiac sympathetic activity did not change after RDN [12]. This is also supported by the lack of change in neurohormonal activation following RDN in the present and in previous studies [16, 17]. Whether this is caused by insufficient denervation or results from a limited overall contribution of renal nerves in determining efferent sympathetic activity could not be assessed because quality parameters for successful RDN are lacking. In the present study we show that the lack of change in renal sympathetic activity may be caused by an inability of RDN to cause a sufficient decrease in afferent sympathetic nerve activity as ^123^I–*m*IBG-uptake did not change significantly.

The amount of published data on renal ^123^I–*m*IBG imaging for the assessment of renal sympathetic innervation is very limited. In addition to our own data, Takamura et al. showed that renal ^123^I–*m*IBG scintigraphy was associated with measurements of muscle sympathetic nerve activity (as a measure of generalized sympathetic outflow) in patients with primary hypertension [18]. In line with our findings, these authors concluded that renal ^123^I–*m*IBG scintigraphy could be a non-invasive clinical tool for assessing renal sympathetic nerve function.

A few limitations of our study merit discussion. Firstly, it remains possible that the modulation of sympathetic nerve activity (SNA) induced by RDN lies below the detection level of ^123^I–*m*IBG. However, it may well be that sympathicolysis is achieved by RDN but that this does not influence BP nor activity of the renin-angiotensin system and ^123^I–*m*IBG parameters. Radiotracer dilution NE spill-over for organ specific assessment of sympathetic nerve activity is an alternative to ^123^I–*m*IBG scintigraphy. Although this technique is considered the gold standard, its application is limited by its invasive nature. Moreover a widespread use of the technique is restricted by the poor availability of the required compounds. Furthermore, ^123^I–*m*IBG is primarily cleared via the kidneys and therefore kidney function may have influenced our data. However, we have shown that both cardiac and renal ^123^I–*m*IBG parameters are not influenced by kidney function [7, 11]. Finally, we were aware of the potential influence of antihypertensive medication (calcium blocking agents, beta blocking agents) that may alter sympathetic drive and thereby uptake of ^123^I–*m*IBG. In two patients, BP lowering medication had to be tapered because of hypotension post RDN. In the remaining patients, however, BP lowering medication and sodium excretion were unchanged during the study period. We therefore feel that changes in antihypertensive medication do not explain the lack of change in the ^123^I–*m*IBG parameters.

In conclusion, we could not observe significant changes in functional kidney denervation as assessed with ^123^I–*m*IBG scintigraphy following RDN with the Symplicity Catheter System. Our data suggest that the lack of BP lowering efficacy in the sham-controlled Simplicity HTN-3 study may be related to lack of procedural effectiveness. In comparison to available clinical tools, renal ^123^I–*m*IBG scintigraphy is minimally invasive and more widely available for clinical use. For future studies, renal ^123^I–*m*IBG scintigraphy may be used as a parameter to assess RDN effectiveness.

## Abbreviations

^123^I–*m*IBG: ^123^I–*meta*-Iodobenzylguanidine
ABPM: Ambulatory blood pressure measurement
BP: Blood pressure
eGFR: Estimated glomerular filtration rate
HTN: Hypertension
MDRD: Modification of diet in renal disease
NE: Norepinephrine
p.i.: Post-injection
PRA: Plasma renin activity
RDN: Catheter based renal sympathetic denervation
ROI: Region of interest
RTH: Resistant hypertension
SNA: Sympathetic nerve activity
SPECT: Single photon emission computed tomography
